# Behavior Change for Youth Drivers: Design and Development of a Smartphone-Based App (BackPocketDriver)

**DOI:** 10.2196/formative.9660

**Published:** 2018-11-26

**Authors:** Ian Warren, Andrew Meads, Robyn Whittaker, Rosie Dobson, Shanthi Ameratunga

**Affiliations:** 1 Department of Computer Science University of Auckland Auckland New Zealand; 2 Waitemata District Health Board Auckland New Zealand; 3 National Institute for Health Innovation School of Population Health, Faculty of Medical and Health Sciences University of Auckland Auckland New Zealand; 4 Section of Epidemiology & Biostatistics School of Population Health, Faculty of Medical and Health Sciences University of Auckland Auckland New Zealand

**Keywords:** smartphone, public health, telemedicine, telemetry

## Abstract

**Background:**

The over-representation of youth in road crash injury and fatality rates is a major public health issue globally. In New Zealand, youth drivers are most vulnerable in the restricted license period when they can drive without the requirement for supervision by an experienced adult. Behavioral change interventions delivered using mobile phone technology to young drivers could serve as a useful mechanism to develop safe driving skills, but this potential remains to be fully explored.

**Objective:**

This study aimed to apply behavioral change principles to design and develop a smartphone-based intervention with the aim of helping youth drivers to develop and hone safe driving skills.

**Methods:**

An iterative process was used to support development of the smartphone intervention. We reviewed behavioral change literature, identifying fundamental principles and exploring use of behavior change techniques (BCTs) in other areas of public health. We engaged with key stakeholders, including young drivers, government agencies, and relevant organizations. We also took into account technology adoption considerations when designing the app.

**Results:**

We developed BackPocketDriver (BPD), an Android smartphone app that uses in-built sensors to monitor and infer driver behavior. The app implements features that were identified during the design process and are traceable to BCTs and theory. A key feature is messaging, which is used to instruct, motivate, educate, and relay feedback to participants. In addition, messaging addresses attitudes and beliefs. Other features include journey feedback summaries, goal setting, achievements, and leaderboards.

**Conclusions:**

BPD’s design rests on a sound foundation of theory and evidence. With explicit links between theory and features, the app aims to be an effective intervention to change and improve youth driver behavior. The next phase of this study is to run a small pilot study to assess BPD’s effectiveness.

## Introduction

Road safety is a significant public health issue worldwide, with approximately 1.3 million fatalities and 20 to 50 million injuries per year, many of which lead to lifelong disabilities. Internationally, road traffic injuries are the leading cause of death among people aged 15 to 29 years [1]. This pattern is also evident in New Zealand where young drivers aged 16 to 24 years are over-represented in crash statistics.

According to recently published data [2] by the New Zealand Ministry of Transport, in 2015, 4% of drivers were aged between 16 and 19 years; however, this age group accounted for 9% of all drivers in minor crashes, 9% of drivers in serious crashes, and 7% of those involved in fatal crashes. Drivers aged 16 to 24 years were involved in 90 fatal crashes, 579 serious injury crashes, and 2608 minor injury crashes. Of these, it was a young driver that was responsible for approximately 80% of the crashes. The social cost for which responsibility was attributed to young drivers was NZ $951 million, 25% of the cost for all injury crashes over the 2015 period [2].

Over time, young drivers tend to become safer. Drivers aged 16 to 19 years are 6 to 8 times more likely to crash than those aged 55 to 59 years, whereas for 20- to 24-year-old drivers, this drops to 3 to 4 times [2]. Particularly, significant factors that cause crashes involving young drivers include speed and alcohol; 53% of young drivers in fatal crashes had alcohol or drugs and/or speed as a crash factor. Other significant factors are losing control of the vehicle and inexperience [2].

Recent and ongoing initiatives have made progress in tackling the youth driver problem. Such initiatives include legislation and graduated driver licensing (GDL), parental involvement to agree on protective limits on teen driving [3], education [4], and training [5]. According to the New Zealand Ministry of Transport’s data, the period when young drivers are at greatest risk of being involved in crashes is when they are on their restricted license [2]. On a restricted license, drivers are subject to conditions, for example, they are not generally permitted to drive after 10 pm or carry passengers. Although legislation and GDL can serve as useful restraints on risky driving behaviors [3], and driver education and training can assist young people to gain the foundational knowledge to obtain their driving license, current initiatives fail to provide young drivers with support for continuous improvement, feedback, and development regarding their driving skills as they begin driving without adult supervision.

Smartphones offer a low-cost sensing platform that enables many facets of driver behavior to be monitored, including speed, acceleration, braking, and steering. These capabilities form a foundation for monitoring, analyzing, and providing feedback on driver behavior [6-9]. Applications of the technology include insurance telematics (Pay How You Drive) [10], detecting impaired driving [11,12], carpooling, and ride sharing—where driver reputation and safety are used to decide who to drive with, eco-driving [13] to reduce pollution, and use of crowdsourced data to identify potential crash-risk areas of the road network [14].

Used in the context of driving, the smartphone, nevertheless, is a double-edged sword. Using a mobile phone while driving is a key contributor to distracted driving, which claims the lives of 5000 Americans annually [15]. Hence, care needs to be taken with any attempt to use smartphones as the basis of a driving intervention. Although moral outrage has not been completely effective in eradicating drink and drive, combining moral arguments with technology shows promise to tackle distracted driving [15]. On one level, an app might block calls and messages while monitoring driver behavior; on another level, it could also augment the monitoring functionality with an array of other features that help to improve driving skills and attitudes.

Interventions in many areas of public health have been based on behavior change techniques (BCTs) [16-18]. A BCT is an observable, nonreducible component of an intervention that is designed to change behavior [19]. There is also strong evidence that particular techniques, for example, setting goals and providing feedback on behavior, have been successful in leading to positive behavioral change among participants [19]. However, the role of BCTs in youth driving interventions is largely unexplored. A notable exception is a review of 6 interventions [20] that found that only a small subset of techniques was employed. In addition, the review identified that the interventions ignored the evidence concerning effective techniques; the techniques actually employed had little overlap with those for which there is evidence that they have been used with success elsewhere.

This study aimed to apply behavioral principles to design and develop a smartphone-based intervention, BackPocketDriver (BPD), with an aim to help youth drivers to develop and hone safe driving skills. Rather than inventing a feature-set based on intuition, we have reviewed behavioral theory, BCTs, and evidence of their effectiveness to develop an informed smartphone app. BPD represents a step toward developing youth-driving interventions that are more theory-led and grounded in evidence. As a result, we expect BPD to be more effective in changing behavior than other apps that are currently available.

## Methods

### Scope

BPD’s development was informed by engaging with key stakeholders, identifying appropriate techniques for behavior change, and relevant design principles for technology-assisted interventions.

This study does not report the outcomes for testing; however, these will be described in the paper on a pilot study of BPD.

### Ethics Approval

The study was given ethics approval by the University of Auckland’s Ethics Committee in February 2016. Informed consent was obtained by participants before participation in the study.

### Stakeholder Engagement

Understanding and incorporating the priorities and preferences of the target audience as well as key stakeholders is important to ensure the success of interventions. Therefore, it is vital to engage with the target population during the design and development process [21].

In the case of BPD, 3 groups were identified as key stakeholders for engagement: young drivers (aged 16 to 24 years), parents of teen drivers, and relevant organizations. The organizations included New Zealand Transport Authority (NZTA), a government entity with a mission to develop a safe land transport system for New Zealand. New Zealand Police were included because of their prevention-first strategy that aims to reduce fatalities and serious crashes. In addition, the Automobile Association (AA) is an independent organization that is actively involved in initiatives for youth driver safety, including license reforms and young driver education.

Engagement with each of the key stakeholder groups was undertaken either in the form of semistructured phone interviews with representatives from the relevant organizations or less formal discussion-based sessions with young drivers and parents. Topics covered with each group of key stakeholders included understanding the issue—causes and implications of crashes involving young people, the level of interest in a smartphone app for safe driving, barriers to engagement with a driving app, preferred functionality and features, and incentives. When interviews were undertaken, these were recorded and transcribed. During sessions with teens and parents, information was captured on flip charts. Teens and parents also completed questionnaires.

### Behavioral Modeling

Several models have been developed to explain behavior. Many of the models share common concepts, and awareness of the fundamental ideas is important in developing BPD.

Drawing on the theory of planned behavior, the dual-process approach, and the prototype willingness model [22], the key concepts are as follows: *target behavior*, *emotions*, *barriers*, *facilitators*, and *willingness* to perform the target behavior. Target behavior is the behavior wanted of participants, contrary to unwanted behavior.

Emotions can influence performance of target behavior. There are 2 emotion types: *anticipated* feelings and *experienced* feelings. Anticipated feelings capture how a person thinks he or she will feel after performing a target behavior. Experienced feelings are the way a person feels at a particular point in time and can influence behavior independently to any intention to perform the target behavior.

Barriers are obstacles that can prevent a person from acting on his or her intentions to perform a target behavior. Intervention design involves helping people to anticipate and overcome particular barriers. Conversely, facilitators make it easier for people to perform target behaviors.

A person’s willingness or intention to perform a target behavior is governed by the following:

- *Norms*. Norms are about what people believe is normal behavior. *Descriptive norms* address the question—“Do people like me perform the target behavior?” People are more likely to perform the target behavior if they believe others like them do. *Injunctive norms* differ in that they are about whether others approve or disapprove of a particular behavior. People are more likely to perform a behavior if they believe others want or expect them to.
- *Control*. This concerns how much control a person believes he or she has over his or her behavior. Control poses the question—“How able am I to perform a target behavior?” Effective interventions build a person’s confidence and capability, contributing to their belief that they can perform a target behavior.
- *Self-identity*. Self-identity addresses how a person’s sense of self aligns with the target behavior. People who align themselves with the wanted behavior are more likely to perform the behavior than if they align with some other identity. Interventions often need to help people change their self-identity or find a solution that fits with their current identity.
- *Attitudes*. *Instrumental attitudes* focus on what a person thinks about a target behavior. For example, people might have the opinion that a target behavior makes them safe, excited, frightened, or bored. On the basis of their thinking, people make a judgment as to whether a behavior is good or bad; where the good outweighs the bad, people are more likely to perform the behavior. *Affective attitudes* are similar but concern how a person *feels* when performing the behavior.

Behavioral models are useful in developing interventions in which they identify a range of psychological elements to address. However, it is often unclear how a particular element can be operated to bring about behavioral change [20]. BCTs offer a solution and define the active ingredients of an intervention.

### Behavior Change Techniques

A BCT is an observable, nonreducible component of an intervention that is designed to change behavior [19]. BCTs can be used in interventions to change one or more psychological determinants of a person’s behavior, including the behavioral modeling elements discussed above [16].

To ensure that the proposed intervention is effective in changing behavior, a review of the literature was conducted to identify relevant BCTs for incorporation into the intervention. We focused on Michie et al’s hierarchical taxonomy comprising 16 categories of 93 distinct techniques [23]. Table 1 introduces each category.

To illustrate a couple of BCTs, *goal setting*, from the *goals and planning* category is concerned with setting short-term goals. To meet a series of goals, a person’s attitude and behavior might change in some desired way. Where a person takes ownership of goal setting, their intrinsic motivation also tends to increase [24]. *Social comparison*, from the *comparison of behavior* category, involves drawing attention to those who exhibit good behavior. In doing so, people are able to compare their own behavior with that of exemplars. This can motivate people to reach or exceed the exemplary behavior.

**Table 1:** Behavior change technique categories.

Category	Description
1. Goals and planning	Setting goals for the target behavior, making plans to achieve goals, and dealing with any barriers
2. Feedback and monitoring	Monitoring progress toward goals and providing feedback to users
3. Social support	Providing social support, from friends, family, colleagues, and professionals to help meet goals
4. Shaping knowledge	Assisting users to better understand their behavior and how to perform target behaviors
5. Natural consequences	Highlighting consequences of performing particular behaviors, enabling users to see that they would regret not changing behavior
6. Comparison of behavior	Comparing participants’ behavior with that of others and leading users to consider whether others approve (norms in a psychological model)
7. Associations	Associating target behavior with positive things and reminding users to perform the behavior
8. Repetition and substitution	Enabling users to practice and develop skills so that target behavior becomes habitual
9. Comparison of outcomes	Allowing users to explore the outcomes of exhibiting or not exhibiting the behavior
10. Reward and threat	Rewarding the target behavior and punishing unwanted behavior
11. Regulation	Easing the task of performing the behavior, for example, by reducing negative emotions that result from the target behavior
12. Antecedents	Understanding what triggers unwanted behavior, taking steps to avoid the triggers, and changing the physical environment
13. Identity	Encouraging users to believe that the target behavior is right for them
14. Scheduled consequences	Arranging a schedule of punishments and rewards for users performing the target behavior and not the unwanted behavior
15. Self-belief	Building user confidence that a participant can perform the target behavior
16. Covert learning	Enabling users to imagine consequences arising from performing a behavior and observing the consequences to others as they perform behaviors

**Table 2:** Behavior change techniques that have generally featured in successful interventions.

Behavior change technique	Description
1.1 Goal setting (behavior)	Set or agree a goal defined in terms of the target behavior
2.2 Feedback on behavior	Monitor or observe behavior and provide feedback on performance of the target behavior
2.3 Self-monitoring of behavior	Establish a method for the person to monitor and record their behavior(s)
3.1 Social support	Advise on, arrange, or provide social support or encouragement for performing the target behavior
4.1 Instruction on how to perform the behavior	Agree or advise on how to perform the target behavior
5.1 Information about health consequences	Provide information about the consequences of performing the target behavior

BCTs have been widely used in many areas of public health. Table 2 describes BCTs for which there is strong evidence that they have been effective in changing behavior for adolescents and adults regarding a broad range of behaviors: obesity, physical exercise, diet and nutrition, and drug use [19]. Each BCT is numbered in Table 2 such that the first digit identifies its category (from Table 1), and the second digit uniquely identifies the BCT within the category.

Developing youth driving interventions that are informed by behavioral change theory has largely been ignored [22,19]. One study [1], however, conducted a review of 6 youth driving interventions to expose any BCTs being used. The interventions included conventional presentations, crash analysis activities, interactive discussions, and a theatrical show with road safety messages. The study found that all of the interventions gave information about consequences and risks (BCT 5.1). The majority of the interventions also demonstrated how to perform target behaviors (a category 6 BCT) and provided feedback on performance of wanted behaviors (BCT 2.2). In 4 interventions, participants were supported in forming intentions (part of category 1 goals and planning) that would help them to perform a target behavior, such as not using a mobile phone while driving. In short, the study exposed that only a narrow set of BCTs were used in the interventions, and there is little overlap between those used and those with strong evidence of effectiveness (Table 2).

At the time of writing, we were not aware of any smartphone-based interventions for youth driving that have been designed with consideration of behavioral change theory. However, later in this paper, we report on popular apps for youth driving and identify features that can be traced back to particular BCTs.

### Design for Technology-Assisted Intervention

Developing technology-assisted interventions is not without challenges. As discussed earlier, participants must be willing to engage in the intervention. With a smartphone-based intervention such as BPD, they must also be *willing* to use the mobile phone technology [25]. In this context, willingness requires that participants *accept* the technology and that they perceive *gains* and little *risk* from its use [25]. Gains can be extrinsic, for example, insurance discounts, or intrinsic, with participants genuinely willing to improve their driving skills. Risks represent potential barriers to use of the technology.

Technology acceptance can further be affected by 4 attributes [25]:

- *Delay discounting*. For youth driving interventions, the benefits—improved driving skills—are likely to come later. With no immediate benefit, youth drivers who discount delayed benefit tend to have a lower perception of the gains associated with engaging with the intervention. This suggests that the technology needs to include appealing features that offer value in the short term and which retain user interest until longer-term gains are evident.
- *Social influence* is concerned with how social groups and peer pressure influence norms, decision making, and behavior. For BPD, features that appeal to youths and youth groups are likely to lead to a more successful intervention. Similarly, features that are perceived by youth as *uncool* are likely to be detrimental to intervention success. Social influence is addressed in behavioral modeling using norms and self-identity.
- *Usability* covers a range of issues, including general user interface (UI) design, but more specifically, for BPD, it is the ease with which the app can be downloaded and used. If the mobile phone needs to be held in a fixed position while driving, necessitating the use of a dashboard mount and a calibration step before each journey, then the app’s perceived usability would be reduced.
- *Attitude*, as discussed earlier in behavioral modeling, concerns an individual’s disposition toward an intervention. Those who are intrinsically motivated or whose motivation can be developed, perhaps extrinsically, are more likely to engage with an intervention.

## Results

### Stakeholder’s Feedback

A summary of the learnings from the stakeholders is presented in Table 3. All 3 groups recognized the youth driving problem and were concerned with the over-representation of road crashes involving young drivers. Limited experience and maturation were identified as key factors, with the Police noting that the majority of fatal crashes are caused by mistakes and inattention. The New Zealand transport agency (NZTA) was interested in the role an app could take as part of a more holistic program to publicize safe driving and to make drivers aware of the effect of their actions on other road users. The Police were supportive of interventions such as BPD that could contribute toward addressing road safety issues. The stakeholder organizations were also more supportive where an app is evidence-based and grounded in (behavioral change) theory. Interest in the BPD concept from young drivers and parents was also positive, notwithstanding potential *risks* to adoption.

Young drivers raised a number of risks relating to privacy. Teens did not want their parents to be able to track their movements or to receive real-time alerts of poor driving behavior. Some teens also raised concerns about the data being made available to authorities and used, for example, to issue speeding infringement notices.

Quality of feedback was an important concern raised by young drivers. Youth drivers wanted reassurance that any feedback would be useful and effective. In addition, they felt that they would be stressed by negative or *nagging* feedback, for example, suggesting that they were a *bad* driver. Similar concerns were voiced about the app being able to consider real conditions, for example, the need to brake heavily to avoid an accident, and subsequently not rating the driving as poor.

Young drivers also expressed *usability* concerns about the effect of using the app on their smartphone’s battery, storage, and mobile data. Liberal consumption of any of these resources would be unacceptable.

The Police identified that youth who are interested in using the app are unlikely to be those who engage in criminal behavior. There exists a correlation between criminality, antisocial behavior, and car crashes, with risk taking and poor decision making being contributory factors. Appealing to this demographic subset, given its *attitude*, therefore poses a challenge. Another attitude-related issue, raised by the NZTA, concerns the potential to subvert the intention, for example, youth using the app for bragging rights and sharing incidents of fast, dangerous, or reckless driving.

Constructive feedback was important to young drivers. Although negative feedback poses a risk, feedback that is encouraging and addresses both good and bad driving, allowing users to discover what they are doing wrong, was viewed as something that young drivers could *gain* from. Similarly, education was also important to youth, suggesting that tips for passing the practical driving test and practice questions for the theory test would be desirable.

Parents were interested in monitoring both routes driven and driving behavior of their teens. Similar to young drivers, parents saw value in the app providing their teens with driver education and instruction. Parents favored automatic deactivation of the phone while driving; however, teens wanted this tempered, for example, to be aware of when a short message service (SMS) text message had arrived but having to stop the vehicle before reading the message.

**Table 3:** Key findings from stakeholder engagement to inform app development*.*

Finding category	Young drivers	Parents of teen drivers	Relevant organizations
Risks to adoption	Threats to privacy; Negative or inaccurate feedback on driving; Battery and mobile data consumption; Excess use of push notification or audio alerts; Cost		App being used as a source of distraction; Appeal of app to most at-risk drivers; Potential to subvert intervention; Cost
Gain enablers	Constructive feedback; Safe driving education; Peer competition	Ability to monitor teens’ driving and behavior; Automated deactivation of phone while driving; Suggestions to improve driving	Sticky intervention; Data analytics based on crowd-sourced data
Incentives	Recognition of achievements; Use of app data as proof of safe driving; Endorsement by relevant organizations, for example, NZTA^a^; Esthetics and ease of use	Integration with licensing process	Material rewards schemes, for example, fuel discounts; Automated starting or stopping of journey monitoring

^a^NZTA: New Zealand Transport Agency.

**Table 4:** Mapping objectives to behavioral elements and behavior change technique categories.

Objective	Behavioral elements	Behavior change categories
1. Improve driving skills	Emotions; Control; Barriers	2. Feedback and monitoring; 4. Shaping knowledge; 7. Associations; 8. Repetition and substitution
2. Strengthen intentions to perform target behaviors	Facilitators	1. Goals and planning; 10. Reward and threat; 13. Identity
3. Increase positive attitudes toward performing target behaviors	Attitudes; Norms; Barriers; Facilitators	3. Social support; 5. Natural consequences; 6. Comparison of behavior
4. Manage self-identity	Self-identity	4. Natural consequences
5. Address the mismatch between perceived and actual driving skills	Facilitators	13. Identity

The AA reported that conventional driver training decays over time. Conversely, a driving app has the potential to remain supportive and of value to youth over time. To do so, it needs to be *sticky* by providing features that retain users’ interest and engagement, for example, material rewards, peer competition, and social comparison. This also helps to combat delay discounting, where the benefit of improved driving skills is seen later and only after a period of participating with the intervention. In addition, to address *usability,* the AA suggested that BPD should automatically start and stop journey monitoring without the need for user involvement, thereby providing for seamless operation and preventing fatigue.

### App Design

BPD has 3 target behaviors for young drivers:

1. To drive within speed limits.
2. To perform maneuvers safely and in a controlled manner.
3. To not use a mobile phone while driving.

These behaviors lend themselves to BPD’s smartphone-based delivery platform because they can be automatically tracked by the smartphone. On the basis of the gathered data, the app can generate tailored responses to help participants develop the wanted behaviors.

We have identified 4 objectives from the target behaviors. For each objective, we have identified the relevant behavioral model elements to operate on. In selecting particular BCTs for each objective, we have considered which BCTs have been proven to work in other interventions. In addition, we have considered which BCT categories are best placed to meet particular objectives. To increase skills, for example, category 8 (repetition and substitution) is appropriate, whereas category 5 (natural consequences) is well suited to changing attitudes [22]. Table 4 presents the objectives, associated behavioral elements, and appropriate BCT categories.

#### Improving Driving Skills

For objective 1, to improve driving skills, BCT category 2 (feedback and monitoring) plays a key role by offering BCTs that can be used to monitor driving behavior and relay feedback to participants. On the basis of feedback, areas to focus on can be identified, enabling participants to practice and improve on these aspects. Category 4 (shaping knowledge) can be employed to assist with improving skills through BCTs that educate participants, for example, by providing instruction on how to perform maneuvers and antecedents to performing the target behaviors poorly. Category 7 (associations) includes BCTs for prompting wanted behavior at particular times. BCTs from category 8 (repetition and substitution) help with honing target behaviors through practice. They also facilitate formation of good habits.

A person’s emotional state can affect his or her driving behavior. As discussed earlier, experienced feelings, such as being upset, can negatively impact performance of target behaviors even when a person has strong intentions and a positive attitude toward the target behaviors. Category 4 is of further value for objective 1 in which it has BCTs that can be used to educate participants in recognizing and managing emotions. Moreover, category 4 can be used to raise participants’ awareness of barriers to performing wanted behaviors, for example, poor time management, drugs and alcohol, tiredness, and phone use. In shaping knowledge, the intervention can suggest how to deal with barriers.

A necessary element to improving driving skills is self-belief—participants must believe that they are capable of performing the target behaviors. BPD can strengthen participants’ self-belief through applying BCTs in category 8. This category includes a BCT for graded tasks, where tasks become more difficult over time. As participants work through a grade or level, they become more proficient and prepared for the next. In terms of driving to speed limits, for example, successive levels might lower the speeding tolerance for achieving a speed-focused goal.

#### Strengthening Intentions to Perform Target Behaviors

Regarding the second objective, to strengthen intentions to perform target behaviors, we recognize that while many participants have a positive attitude toward the target behaviors, without goals they might lack the impetus to engage and develop the wanted behaviors. BCT category 1, goals and planning, is appropriate to draw on as it provides BCTs for participants to set and track progress with goals associated with target behaviors.

Category 10, rewards and threats, can also be used to incentivize participants. Social rewards recognize that participants have performed a target behavior well and provide a sense of achievement. Similarly, category 13, identity, includes a role-modeling BCT where a participant can be elevated to a role model after performing well in a target behavior. This can bring a sense of kudos to the participant, fostering their motivation and engagement.

#### Increasing Positive Attitudes Toward Performing Target Behaviors

Although attitudes of many young people align closely with safe driving, there are others who hold less positive attitudes toward BPD’s target behaviors. Hence, for some participants, the intervention needs to change their thinking (instrumental attitude). This is the motivation for objective 3 to increase positive attitudes toward performing target behaviors. BCT category 5, natural consequences, includes BCTs that can be applied to help participants see the consequences of performing wanted or unwanted behaviors. Related to consequences is the notion of anticipated regret, which involves having a participant think about how they would feel if they did not change their behavior and continued to perform an unwanted behavior, for example, speeding. Thinking through an undesirable outcome may contribute to change in instrumental attitude.

Descriptive norms influence attitudes. To show that it is normal for other young people to perform the target behaviors, BCTs from category 6, comparison of behavior, can be used. Social comparison involves bringing to the attention of participants other participants who they consider to be part of the same social group and who are performing the target behaviors well. Category 6 also includes BCTs for addressing the approval of others (injunctive norms). In addition, where celebrity figures who are respected by young people endorse the target behaviors, this might also contribute to changing attitudes and meeting objective 3.

BCT category 3, social support, is also appropriate to consider for the third objective. The BPD app could include social networking functionality allowing participants to support one another in developing the target behaviors. Another supportive role for BPD would be to address barriers to performing the target behaviors. Barriers include peer pressure and triggers, for example, racing or using mobile phones while driving. The app could deliver advice on how to deal with such barriers.

#### Managing Self-Identity

Objective 4, to manage self-identity, recognizes that a person’s attitude might be opposite to the target behaviors. For BPD, *boy racers* are an obvious group that is unlikely to view positively the target behaviors of driving within speed limits and conducting maneuvers safely. To address such groups, category 5 BCTs can be used to help change attitudes, similarly to their role in objective 3. Realistically, however, a more effective approach might be to complement use of natural consequences BCTs with a solution that allows boy racers to become aware that they can satisfy their need for speed and thrill seeking through other means.

#### Addressing the Mismatch Between Perceived and Actual Driving Skills

The final objective concerns the mismatch between perceived and actual driving skills. Young drivers tend to overestimate their safety margin, resulting in more risk taking [26] and a heightened optimism bias that leads them to underestimate the likelihood of negative outcomes from unwanted driving behaviors [22]. For example, young drivers might believe that using a mobile phone while driving is dangerous and choose to protect themselves by not doing so when driving on motorways; however, they may still be prepared to take the risk when driving around town [22]. Although BCT categories discussed earlier, notably 1 and 2 for setting goals and receiving feedback, can help to highlight to a driver that their skills are not as good as they think they are, BPD could also expose their incompatible beliefs, for example, thinking that it is not risky to use a mobile phone while driving in an urban environment. BCT category 13’s incompatible belief serves this purpose.

Having identified the subset of BCT categories that are applicable to BPD, Table 5 introduces particular techniques from the categories as deemed relevant to this intervention. In each case, possible application of the technique in the context of BPD is described. The BCTs for which there is strong evidence that they have led to behavior change elsewhere, discussed earlier, and presented in Table 2, are shown in italics.

**Table 5:** Relevant behavior change techniques (BCTs). There is strong evidence that the BCTs shown in italics (outlined in Table 2) have generally featured in successful interventions.

Behavior change technique	Description
*1.1 Goal setting (behavior)*	Mutually agree on short-term goals to be achieved, such as “This week I will brake more gently.”
1.2 Problem solving	Prompt participants to analyze behaviorally influencing factors and develop strategies for overcoming barriers. For example, “So it seems you’ve been having trouble with your speed. How do you think you could try to change that next time you go out?”
1.3 Goal setting (outcome)	Facilitate longer-term goals, such as “Be a safe driver,” “Get my full license,” and “Avoid accidents.”
1.4 Action planning	Prompt participants to plan their driving, including factors such as context, frequency, and duration.
1.5 Review behavior (goals)	Review behavioral goals together with the participant and consider modifying them based on progress. For BPD^a^, goals can be reviewed and modified by the app.
2.1 Monitoring without feedback	Record behavior with the participant’s knowledge. Driving behavior data captured by the app could be made available to a participant’s parents. The knowledge that their driving behavior is being observed can influence their behavior.
*2.2 Feedback on behavior*	Monitor and provide informative feedback on performance. BPD could provide feedback in terms of poor driving behavior, suggestions on how to improve, and recognition of good behavior.
*2.3 Self-monitoring of behavior*	Establish a method for participants to monitor their own behavior. BPD could provide the ability to review earlier feedback and to identify behavioral trends.
2.7 Feedback on outcomes	After periods of prolonged safe driving, BPD might inform participants that they are now statistically less likely to be involved in an accident than when they started the intervention.
*3.1 Social support (unspecified)*	Arrange for participants to receive support from others. In BPD, this could take the form of a social network connecting participants and friends.
*4.1 Instruction on how to perform a behavior*	Provide advice on how to perform a behavior. BPD could present how-to messages, describing techniques, and practices that help participants to perform the target driving behaviors.
4.2 Information about antecedents	Provide information about situations, events, or emotions likely to cause poor performance of the target driving behaviors.
*5.1 Information about health consequences*	Provide information about the positive or negative health consequences of wanted or unwanted behavior. BPD could deliver messages concerning the benefits associated with target behaviors.
5.2 Salience of consequences	Use methods to emphasize consequences for *5.1*, for example, having BPD display images of car wrecks and devastated loved ones.
5.5 Anticipated regret	Have participants imagine how regretful they would feel if they perform unwanted behavior, for example, speeding and something negative happens.
6.2 Social comparison	Draw attention to performers of good behavior to allow comparison with a participant’s own performance. For example, BPD could maintain a *leaderboard* allowing participants to see how well others are driving.
6.3 Information about others’ approval	Provide information about what other people think about good and bad behavior. BPD could provide informational messages about the negative social perception of unsafe drivers (or vice versa).
7.1 Prompts or cues	Introduce stimuli to encourage good behavior. BPD might provide NFC^b^ sticker tags that participants can place in their vehicles to remind them to use the app and put their phone away.
8.3 Habit formation	Prompt rehearsal and repetition of good behavior in the same context repeatedly, so the context elicits the behavior. Having finished using BPD, participants should continue to perform the target behaviors they have developed habitually.
8.7 Graded tasks	Set easy tasks and then gradually make them harder as participants improve. BPD could offer goals at varying difficulty levels and ensure that participants make progress through the more challenging goals.
10.1 Material incentive (behavior)	Inform participants that a material reward ( eg, money or vouchers) will be given in exchange for demonstration of the target behavior. BPD might seek partnership with businesses and organizations to provide such rewards.
10.4 Social reward	Similar to 10.1, but rather than a material incentive, the incentive would enhance a participant’s standing in some way. Performing target behaviors in BPD could earn participants achievements.
10.11 Future punishment	Inform participants that punishment or loss of reward occurs if poor behavior continues. BPD might simply raise awareness of legal or social punishments in response to detecting prolonged poor driving behavior.
13.1 Identification of self as role model	Inform participants that their good behavior is an example to others. BPD could promote demonstrably safe drivers to others, offering a level to aspire to.
13.3 Incompatible beliefs	Draw attention to discrepancies between current or past behavior and self-image to create discomfort. BPD could use messaging to highlight differences in actual versus perceived driving skills and incompatible beliefs over driving practices.

^a^BPD: BackPocketDriver.

^b^NFC: near field communication.

In addition to the BCTs for which there is strong evidence that they have led to behavioral change in other interventions, BCTs 4.2 information about antecedents, 5.5 anticipated regret, 7.1 prompts or cues, 8.3 habit formation, and 13.3 incompatible beliefs are seriously worth considering because they are founded in behavioral change theory [22]. Others, including 6.2 social comparison, 6.3 information about others’ approval, 10.4 social reward, and 13.1 identification of self as role model, currently lack evidence but appear interesting and relevant to BPD. Although there is no evidence yet to support BCTs 6.2, 6.3, 10.4, and 13.1, it is nevertheless valid to apply them in our intervention to determine their success in the context of youth driving.

### App Development

On the basis of the design considerations, as discussed above, we have identified several features for the BPD smartphone app. The features are informed based on the selection of BCTs that are appropriate for the intervention. Each feature is described below.

#### Achievements

BPD uses social rewards *(BCT 10.4)* to reward participants who exhibit the target behaviors. Essentially, participants accrue points over time. Recognizing achievement was also a feature wanted by the target demographic.

#### Goal Setting

Goals are fundamental to BPD *(BCT 1.1)*. Goals are presented in the form of “I will...” statements to promote the user’s sense of attachment to the task, for example, “This week I will try not to steer jerkily.”

On the basis of a participant’s prior driving performance, BPD suggests particular goals that users can modify in terms of difficulty. Users are encouraged to choose more difficult goals as their driving performance improves *(BCT 8.7)*.

#### Journey Summaries

At the conclusion of each driving episode, users can review their performance *(BCT 1.5)*. Information displayed includes a map with the participant’s route, highlighting incidents of good driving behavior, and others where driving can be improved. Feedback is provided *(BCT 2.2)* to include advice on how to modify behavior to reach goals. Where goals are being achieved, suggestions on more challenging goals are offered *(BCT 8.7)*.

In addition to postjourney feedback, users have the opportunity to view feedback on previous journeys *(BCT 2.3)* and are prompted to use this feature if they have not used it for a while. This encourages them to view their progress over time.

#### Messaging

BPD makes liberal use of messaging as a means to meet many of the intervention’s objectives introduced earlier. Messages serve many purposes, including providing information relating to instruction, consequences, antecedents, anticipated regret, feedback, other’s approval, and incompatible beliefs. Table 6 lists a selection of messages to illustrate the type of content participants can expect to see and shows the mapping to objectives and BCTs.

Messages are generally framed in terms of gain as opposed to loss, which has been shown to be more effective in leading to behavioral change [20]. In addition, the app generates messages that are specific to participant behavior. Again, messages linked to actual behavior are more effective in facilitating behavior change than those that simply offer general encouragement to drive safely [20].

BPD displays messages to users at different times in response to different stimuli. *Prejourney messages* are shown whenever the user starts a new journey. Prejourney messages remind the user of their goals for the journey in an encouraging manner.

*Postjourney messages* offer feedback on how well a user has driven *(BCT 2.2)*. Positive messages are shown if a user has done particularly well, for example, *“* You’ve kept to speed limits today! Well done!*”* In other cases, encouragement with constructive criticism and tailored advice is offered, for example, *“* Well done on your journey today, but we noticed it was a little rough at times. Try to allow more time to stop the vehicle in the future so you don’t have to slam on the brakes*” (BCT 4.1)*. Where prolonged periods of poor behavior are observed, message content discourages continued poor driving *(BCT 10.11).*

*Daily messages* are sent to educate participants, for example, the messages derived from BCTs 4.1 and 4.2 in Table 6. They may also include content targeting attitudinal change, where appropriate, and based on behavior data gathered by the app, for example, the messages associated with objectives 3 and 4 in Table 6. Daily messages additionally prompt self-monitoring *(BCT 2.3)* by reminding the user of BPD’s functionality, for example, *“* Did you know you can review your past journeys by accessing the Journey History from the main menu?*”*

#### Friends

Users can connect with elected friends who are also using the app, facilitating social support *(BCT 3.1)*. This provides users with the opportunity to share achievements, statistics, and commentary with others.

#### Leaderboards

The app operates a leaderboard with which users can compare their own progress with that of other participants. This facilitates social comparison *(BCT 6.2)* and promotion of good drivers with role models *(BCT 13.1).* Leaderboards are a popular gamification element and have been shown to be effective in incentivizing user engagement in other apps [27]. Leaderboards also offer a means to peer competition, which is attractive to the target demographic.

#### Detection of Driving Conditions

Smartphones are capable of detecting many aspects of the driving environment, for example, time of day, type of road, and prevailing weather. Detecting driving conditions is a feature that enables automated generation of a driving log, including hours spent driving on different road types. Such logs are a requirement for learner drivers in some jurisdictions. Automated logging protects against the possibility of fraudulently entered manual log entries. As part of the stakeholder engagement, the NZTA viewed logging positively.

#### Journey Detection

BPD implements near field communication (NFC)–initiated journey monitoring. Participants stick an NFC tag on their dashboard and swipe their phone over the tag to commence monitoring. The tag additionally serves as a cue *(BCT 7.1)*, reminding users to put away their phone and drive safely. BPD automatically detects cessation of vehicle movement for a prolonged period of time, stopping monitoring, and recording that the driving episode has concluded. For mobile phones that are not NFC-enabled, users begin monitoring by pressing a button within the BPD app.

An alternative approach would be to automatically detect, without any participant action, the start of a journey. This would promote usability and would also ensure that all journeys are monitored. However, automated detection requires the device’s accelerometer to be activated at all times, which causes significant battery drain. As this sort of interference was seen as a risk to adoption by the target demographic, BPD does not implement the automated detection.

#### Additional Driving Behavior Detection

Detecting driver behaviors other than speed and smoothness, for example, following (stopping) distances, is possible by using additional mobile phone sensors. This is discussed further in the Discussion section.

#### Rewards Scheme

By offering material incentives *(BCT 10.1)* in exchange for demonstrating good driving behavior, some users will be more extrinsically motivated to achieve their goals. However, we focus on intrinsic motivation only in this study because, as noted earlier, intrinsic motivation tends to lead to better long-term behavior change [24].

#### Parental Interface

Parents showed interest in being able to monitor their children’s driving behavior, although the target demographic viewed parental involvement as a risk to adoption and use of BPD. A parental interface has not been implemented.

**Table 6:** Sample messages derived from objectives and behavior change techniques (BCTs).

Message	Objective	BCT^a^
*Remember to take your foot off the accelerator prior to cornering so you don’t need to brake so suddenly.*	1	4.1
*Distractions like eating, changing music, and passengers can make you unsafe when driving. Try to minimize distractions as much as possible.*	1	4.1
*Did you know that driving while tired is as risky as driving while intoxicated?*	1	4.2
*You’re much less likely to be involved in an accident if you keep within the speed limit.*	1	4.2
*In a bad mood today? Don’t take it out on the road. Take a few deep breaths before turning the car on.*	1	4.2
*Allow enough time for your journey so that you don't feel the need to speed.*	1	4.2
*You’re just about to start driving. It’s now time to put your phone away for today’s journey.*	1	7.1
*Drive to the speed limits and you’ll avoid demerit points. You’ll keep your license and enjoy the freedom from driving.*	3, 4	5.1
*How would you feel if you crashed because you lost control of your car? How would it affect your friends?*	3, 4	5.5
*Kids want you to share the roads with them safely. Slow down around schools and watch out for kids playing.*	3, 4	6.3
*Does a mate want you to race with them? Weigh it up—is the thrill of a race that will be over before you know it worth the risks?*	4	5.5
*Feeling the need for speed? Arrange a go-karting track session with your mates. That’s the way!*	4	4.2
*Using a mobile phone on the motorway would be crazy! Did you know it’s just as risky using a mobile phone around town?*	5	13.3

^a^BCT: behavior change technique.

**Table 7:** Feature wish list for BackPocketDriver (BPD).

Feature		Behavior change techniques applied
**Must haves**		
	Location and speed detection^a^	—^b^
	Acceleration, braking, and turning detection^a^	—
	Phone usage detection^a^	—
	Achievements	BCT 10.4 *(social reward)*
	Goal setting	BCT 1.1 *(goal setting);* BCT 8.7 *(graded tasks)*
	Journey summaries	BCT 1.5 *(review behavior goals);* BCT 2.2 *(feedback on behavior);* BCT 2.3 *(self-monitoring)*
	Messaging	BCT 2.7 *(feedback on outcomes);* BCT 4.1 *(instruction);* BCT 4.2 *(info about antecedents);* BCT 5.1 *(info about health consequences);* BCT 5.5 *(anticipated regret);* BCT 6.3 *(info about others’ approval);* BCT 13.3 *(incompatible beliefs)*
**Should haves**		
	Journey detection	BCT 7.1 *(prompts or cues)*
	Friends	BCT 3.1 *(social support)*
	Leaderboards	BCT 6.2 *(social comparison);* BCT 10.4 *(social reward);* BCT 13.1 (*identification of self as role model*)
	Detection of driving conditions	—
**Could haves**		
	Additional driving behavior detection	—
**Nice to haves**		
	Rewards scheme	BCT 10.1 *(material incentive)*
	Parental interface	BCT 2.1 *(monitoring without feedback)*

^a^Necessary for target behaviors: 1 *(drive within speed limits);* 2 *(perform maneuvers safely and in a controlled manner)*; 3 *(not use a mobile phone while driving)*.

^b^Not applicable.

Table 7 summarizes the feature set for BPD showing the linkage between features and BCTs. Software development followed an agile development process. Target users contributed to the development of wireframe models of BPD’s UI, and software development progressed iteratively taking into account user feedback. Feature development was prioritized using the Must have, Should have, Could have, Won’t have (MoSCoW) [28] method. In implementing the app’s UI, Android’s material design guide [29] was applied to ensure conformance with established principles and patterns for implementing UIs on small mobile devices.

### App Implementation

**Figure 1:**
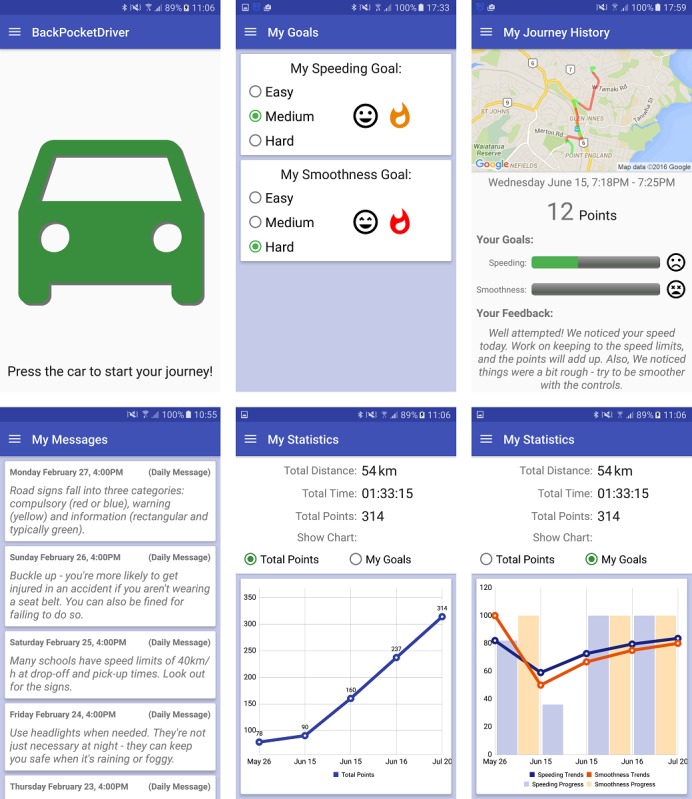
Screenshots of the BackPocketDriver app.

Figure 1 shows several aspects of the BPD app. To start a journey, users either press the car-shaped button on the app’s home screen (a) or swipe an NFC tag.

Before starting a journey, users choose goals to work toward, for example, Figure 1 (top center) where a user has chosen a moderate speeding goal and a more challenging smoothness goal. Once a journey has been completed, the smartphone app sends the journey data to the BPD Web service, accessible via a Wi-Fi link, over the internet.

Having processed the data, the Web service sends feedback to the BPD app. Upon receipt, a notification appears in the device’s notification tray. Users click the notification or navigate back into the app to view the journey summary (Figure 1, top right). The screen shows a map with their route, which is color-coded according to areas of good (green) or poor (red) driving behavior. Progress bars and icons detail how close a user was to achieving their goals for the journey, whereas a feedback message relays further information. Trends can be viewed such as those in Figure1 (bottom center and bottom right), which allow users to view their progress over time.

All messages generated by BPD are viewable at any time on the screen shown in Figure 1 (bottom left). This includes journey-related messages in addition to daily messages that are sent via push notification to the device by a Web service. Messages are generated so as not to unnecessarily repeat content and to provide a fresh user experience.

The next step is to run a small study to assess BPD’s potential for effectiveness in developing safe driving skills among youth. A before-after study is currently underway involving 20 participants, aged 16 to 24 years, and on their restricted or full license. Participants are monitored using a minimal BPD app for 1 month to classify their driving behavior. They then switch to the full BPD app that includes the behavioral-change feature set for a second month. Following the study, any change in driving behavior will be identified based on app-generated data, and participants will have an opportunity to provide feedback on the intervention.

## Discussion

### Context

This study outlines the design of a smartphone-based intervention for developing safe driving skills among youth drivers. Although other researchers have investigated the use of smartphone technology in monitoring driver style and behavior, work that has sought to *improve* driving skills of youth is very limited. Moreover, we are not aware of other work that has taken an approach rooted in behavior theory, evidence, and BCTs to inform design of a smartphone intervention.

### Related Work: Existing Driving Apps

There is a plethora of driving-related apps available from app stores. Many apps target a particular aspect such as preparing for licensing theory tests (eg, Theory Test Kit and New Zealand Driving Theory Test), logging journeys (eg, DrivePad), and blocking messages, calls, and notifications during driving (eg, DriveMode, Shut Up and Drive, and Safe Ride). There are also apps that, similar to BPD, aim to assist young people to develop safe driving practices. We have selected 6 popular apps that appear to have overlapping objectives with BPD and which offer more than simple blocking or logging functionality.

For each app, we have examined its features and identified any BCTs to which features are attributable. Table 8 shows that BCTs are organized into 4 categories: the BCTs listed in Table 2 for which there is strong evidence that they have led to behavioral change, those that lack strong evidence to date but are rooted in behavioral theory, others that we identified earlier as interesting for a youth driving intervention, and others that we have not viewed as fundamental to BPD but are linked to the surveyed app(s).

LifeSaver is a blocking app that automatically silences a user’s smartphone on detecting driving. Journey feedback (BCT 2.2) is limited to reporting on the unwanted behavior of using the phone, for example, to text while driving. At the end of a journey, the app displays a percentage score where 100 indicates that the user did not use their phone while driving. LifeSaver supports a family view, which is essentially a leaderboard that ranks the family members according to their scores. As the leaderboard shows each family member’s score, teens are aware that their parents are monitoring them (BCT 2.1). Through location tracking of family members, the app facilitates social support (BCT 3.1) as users can see when their family members are driving and defer calling them until they have finished their journey.

TrueMotion Family is similar to LifeSaver in that it is also a family-oriented app with a leaderboard that publishes each family member’s driving score. In addition to phone use, for example, texting and calling, TrueMotion Family also factors aggressive driving and speeding to generate a user’s score. The app pinpoints unwanted behavior events on a map allowing the user to see where the events occurred; it also allows users to review their driving behavior over time (BCT 2.3).

**Table 8:** Behavior change technique feature matrix for popular youth driving apps.

Behavior change technique		LifeSaver	TrueMotion Family	Mojo	DriveSmart	EverDrive	Steer Clear
**Behavior change techniques used in successful interventions**							
	1.1 Goal setting (behavior)						
	1.3 Goal setting (outcome)						✓
	2.2 Feedback on behavior	✓	✓	✓	✓	✓	
	2.3 Self-monitoring of behavior		✓		✓	✓	
	3.1 Social support	✓	✓				✓
	4.1 Instruction on how to perform the behavior			✓			✓
	5.1 Information about health consequences						
**Behavior change techniques grounded in behavioral theory**							
	4.2 Information about antecedents						
	5.5 Anticipated regret						
	7.1 Prompts or cues						
	8.3 Habit formation	✓	✓	✓	✓	✓	
	13.3 Incompatible beliefs						
**Behavior change techniques that appear relevant to a youth driving intervention**							
	6.2 Social comparison	✓	✓	✓		✓	
	6.3 Information about others’ approval						
	10.4 Social reward	✓	✓	✓		✓	
	13.1 Identification of self as role model	✓	✓	✓		✓	
**Other Behavior change techniques**							
	1.8 Behavioral contract						✓
	2.1 Monitoring of behavior by others without feedback	✓	✓	✓			
	10.2 Material reward			✓	✓		✓
	10.11 Future punishment				✓		

Mojo, similar to LifeSaver and True Motion Family, employs a leaderboard that ranks teens among their friends based on points earned while driving. As with LifeSaver, scoring is based on unwanted phone use alone. LifeSaver breaks down feedback, for example, providing a count of swipes and taps that a user makes on their phone while driving. Mojo differs by employing material rewards (BCT 10.2). Users who have amassed high scores are invited to spin a wheel for the chance to win a voucher. Mojo’s feedback, rather than being limited to a score, also offers tips for improving behavior. For example, if the user has been making calls while driving, Mojo displays a message to tell the user that they will improve their safety and score by not making calls during future journeys.

DriveSmart monitors driver’s behavior and generates a percentage score based on their braking, cornering, and speeding. Similar to TrueMotion Family, DriveSmart plots driving events on a map and allows users to review their behavior over time. Similar to Mojo, DriveSmart has rewards partners and can offer material rewards in exchange for good driving behavior. Unlike the above apps, DriveSmart does not offer any collaboration features such as a leaderboard; instead, it is intended to be used by an individual and not in a group context. As part of feedback, DriveSmart uses loss-framed messages, alerting users to future punishment (BCT 10.11), for example, for speeding.

EverDrive has a feature set similar to TrueMotion Family. It monitors a driver’s acceleration, braking, cornering, speed, and phone use. Instead of providing feedback through percentage scores, it uses a 5-star scheme.

Unlike the above apps, Steer Clear does not monitor or provide feedback on driver behavior. It includes logging functionality that allows individuals to record their driving hours in different conditions. In addition, it has unique features: a behavioral contract (BCT 1.8), goal setting (BCT 1.3), and videos to share experiences of other users, a form of social support (BCT 3.1). When a user starts using the app, they make a pledge to drive safely; the pledge forms the basis of a behavioral contract. In using Steer Clear, users work toward the outcome goal of completing the set of Steer Clear modules. Once complete, users are eligible for insurance discount (BCT 10.2).

Table 8 reveals that the surveyed apps use quite a narrow band of BCTs. They generally provide feedback (BCT 2.2) and through monitoring and feedback, they help with forming good habits (BCT 8.3).

Many of the apps are group oriented involving family members and/or peers. They include a leaderboard feature, and the way that leaderboards are used is effective in exercising several BCTs. The leaderboards allow social rewards (BCT 10.4) in the form of stars and percentage scores to be publicized to the group, facilitating social comparison (BCT 6.2). They also enable higher scorers to identify themselves as role models (BCT 13.1). In addition, the leaderboards make users aware that they are being monitored by other group members in a way that does not involve feedback (BCT 2.1). Of these, BCTs 10.4 and 13.1 are particularly appropriate for strengthening intentions to perform wanted driving behaviors.

Of the apps that offer feedback on behavior, they do so in the form of a numeric score. Only Mojo and DriveSmart include textual feedback to supplement scores, and even here, the messages do not address health consequences, antecedents, anticipated regret, or incompatible beliefs—that either are proven or theoretically informed BCTs. Furthermore, none of the apps employ goal setting for behavior (BCT 1.1), which is a proven BCT. Similarly, instruction on performing wanted behaviors (BCT 1.4), another BCT for which there is strong evidence that it is effective, is employed very sparsely.

The apps have limited support for increasing positive attitudes toward wanted driving behaviors. The leaderboard feature, linked to BCT 6.2 (social comparison), can help address norms and demonstrates to a teen that others in their social group do exhibit the wanted behaviors. However, many of the other BCTs discussed earlier for addressing attitudes, managing self, and dealing with the actual or perceived skills mismatch are not associated with the surveyed apps’ features. Hence, it seems unlikely that the surveyed apps can lead to long-term behavioral change.

### Related Work: Monitoring Driver Behavior

In recent years, much work has been conducted to validate use of smartphones in providing a low-cost sensing platform and to supersede the older in-vehicle data recorder (IVDR) units that necessitate a fixed installation [8].

Today’s smartphones include inertial sensors that enable smartphone driver support systems (SDSS) to detect driving events such as acceleration, braking, turning, and lane changing [7,9,30-32]. SDSS typically score a driver’s behavior [7] or classify it in some way, for example, passive or aggressive and risky or safe [33]. Other apps have a narrower focus, for example, detecting events that suggest when a driver is driving under the influence of alcohol [11] that complement yet other apps concerned with drink and drive prevention [34]. SDSS offer high levels of accuracy, for example, with rates in excess of 90% for correctly classifying driver behavior [7,11,31,33].

Beyond a smartphone’s inertial sensors, other in-built sensors include cameras and microphones that are being used to detect whether drivers are drowsy or distracted. CarSafe uses both the forward- and rear-facing cameras to monitor the driver’s face and eyes along with the road ahead [12]. CarSafe detects and alerts drowsy drivers and warns them of events such as straddling the center line. DriveSafe [35] similarly warns drivers when they appear to be distracted or drowsy and, additionally, makes use of the smartphone’s microphone, for example, to identify cases where the driver has turned without using the indicators (which are assumed to emit an audible signal when used).

### Related Work: Youth Driving Interventions

In an early SDSS study [36], an app was developed to warn drivers in real time of speeding events and upcoming speed zone changes. Subsequently, and in the absence of any behavior change, the app sent text messages to participants to encourage them to reduce their speed. With 16 teen driver participants, the study found that use of the intervention resulted in a drop from 31% to 18% in speeding incidents. Other studies [37-39] involving use of IVDR systems have similarly reported that monitoring and feedback does improve youth driving behavior. Moreover, an IVDR-based study involving 92 youth drivers found that teen coaching for 6 months is an insufficient period; when withdrawn, incidents of poor driving behavior, having previously declined, began to rise [40].

Parental involvement is a contentious issue for SDSS. Key findings for IVDR systems that involve parents, for example, [37,38,40], are that these interventions can provide useful and objective information to parents concerning teen driving behavior. Where teens believe that their parents might (but will not necessarily) review their driving, they tend to drive more safely. This is consistent with BCT 2.1, monitoring without feedback. Acceptance of parent-focused interventions is mixed because of issues of privacy and trust. In addition, systems that were evaluated by randomized controlled trials did not lead to a reduction in crash rates. Finally, parental involvement can be used to contribute to an intervention, but it is unlikely to be effective without other intervention elements [3]. We refer the readers to Curry et al’s study [41] for more details on parent-focused interventions.

Gamification, the use of game elements in nongame contexts [42], is largely unexplored in SDSS for youth. Gamification uses extrinsic motivators to increase intrinsic motivation [27]. One app that employs gamification does so to encourage young drivers to undertake supervised driving in a range of conditions to improve their driving skills [43]. On the basis of a small study of 25 drivers, a gamified version of the app was found to be more enjoyable and motivating than a conventional nongamified version. Although the gamified app did not lead to behavioral change, it is likely to lead to greater adherence to an intervention.

With mobile phones known to be a source of distraction when driving, the role of SDSS in blocking incoming calls and text messages has been investigated. A study involving teen drivers [44] found that blocking did reduce the number of calls made and the number of text messages sent while driving. In particular, the intervention reduced impulsive calling and texting. However, the study also found subjective data showing that users tried to work around the blocking functionality, for example, by using a friend’s phone while driving.

### Principal Findings

BPD’s feature set has been derived through the application of behavioral theory and BCTs and consideration of the evidence relating to BCT effectiveness elsewhere. Beyond monitoring and classifying driver behavior, which is where much of the existing work in SDSS stops, BPD’s design includes a suite of features that have a clear mapping to distinct BCTs. A key feature is postjourney feedback. Messaging is also employed for many purposes: to instruct, motivate, educate, and relay feedback to participants and to address participants’ attitudes and beliefs. BCTs and gamification are complementary; BPD’s design combines elements of gamification with BCTs in offering goal setting and review and achievements and leaderboards. Leveraging the smartphone sensor platform, the design also allows for monitoring of additional facets of driving behavior and automated detection of driving conditions.

BPD’s design has also taken into account technology acceptance considerations. The app enables youth to perceive gains through constructive feedback, education, and social comparison or competition. These features further contribute to addressing delay discounting and social influence in that they help to retain user engagement and interest over time, not only for individuals but also for peer or social groups.

Key risks have been mitigated. Unlike other SDSS and IVDR systems and apps, BPD does not present percentage scores or include raw or absolute figures for acceleration and speed when providing feedback. This ensures that BPD cannot be used to subvert the intervention, for example, by teens using the app to record and share race times and dangerous driving events. In addition, BPD allows users to control how they share their data in the interests of privacy. Moreover, the app conserves device resources—using no mobile data and minimizing power consumption—so as to have minimal perceived effect on users’ smartphones for daily operation. Furthermore, BPD does not offer real-time feedback, contrary to many SDSS and IVDR systems. This is both to avoid distraction, which has been linked to real-time feedback in other studies [36,41] and to conserve mobile data.

Regarding usability, the app is easy to use. It requires no calibration before use, and during a journey the smartphone can be kept in the driver’s pocket (the app functions accurately without requiring a fixed dashboard mount). The app does not provide a completely seamless experience in that it does not detect the start of a journey and begin monitoring automatically. Implementing this feature would increase the risk that youth would not use the app because the necessary power-draw would interfere with the normal operation of the smartphone. This represents a conflict between requirements; a key conflict among stakeholders is the issue of parent involvement versus youth privacy. In developing BPD, we have sought to minimize risks to intervention adoption.

BPD is complementary to fundamental research aimed at understanding the underlying issues that contribute to youths’ over-representation in crashes. In Shope et al’s study [45], a framework has been developed that identifies 7 categories of influence on teen driving behavior: driving ability, developmental factors, behavior factors, personality, demographic, and perceived and driving environments. Other research has targeted particular influences, for example, risk perception and sensation seeking [46]. As a technology-based intervention, BPD has much potential to address some influences, for example, driving behavior and ability. It could serve as part of a holistic approach to supporting youth driving; such comprehensive approaches necessarily require buy-in from many factions, including government, researchers, public health practitioners, parents, teens, and auto industries [3].

### Conclusions

BPD is a smartphone-based intervention that aims to improve driving skills in youth. Critically, BPD’s design has been informed by behavioral theory and behavioral change expertise. Stakeholder feedback and technology acceptance considerations have also been factored into the design. Having implemented the app on a sound theoretical foundation, the next step is to evaluate its potential to be effective in changing youth driving behavior. A small study involving 20 youth participants is currently underway, and we expect to report on the results in the near future.

## Abbreviations

AA: Automobile Association
BCT: behavior change technique
BPD: BackPocketDriver
GDL: graduated driver licensing
IVDR: in-vehicle data recorder
NFC: near field communication
NZTA: New Zealand transport agency
SDSS: smartphone driver support system
SMS: short message service
UI: user interface
